# Circulating irisin levels in patients with MAFLD: an updated systematic review and meta-analysis

**DOI:** 10.3389/fendo.2024.1464951

**Published:** 2024-12-17

**Authors:** Chenglu Shen, Kaihan Wu, Yani Ke, Qin Zhang, Shuaihang Chen, Qicong Li, Yuting Ruan, Xudan Yang, Shan Liu, Jie Hu

**Affiliations:** ^1^ The First Clinical Medical College of Zhejiang Chinese Medical University, Hangzhou, Zhejiang, China; ^2^ School of Basic Medical Sciences, Zhejiang Chinese Medical University, Hangzhou, Zhejiang, China; ^3^ The Second Clinical Medical College of Zhejiang Chinese Medical University, Hangzhou, Zhejiang, China; ^4^ Department of Clinical Evaluation Center, The First Affiliated Hospital of Zhejiang Chinese Medical University, Hangzhou, Zhejiang, China; ^5^ Department of Infectious Diseases, The First Affiliated Hospital of Zhejiang Chinese Medical University, Hangzhou, Zhejiang, China

**Keywords:** non-alcoholic fatty liver disease, metabolic dysfunction-associated fatty liver disease, irisin, systematic review, meta-analysis

## Abstract

**Objective:**

Current research suggests that irisin is closely linked to the pathogenesis and progression of metabolic dysfunction-associated fatty liver disease (MAFLD). This systematic review and meta-analysis updates our previous meta-analysis and further explores the relevance between circulating irisin levels and MAFLD.

**Methods:**

Nine databases (PubMed, EMBASE, Cochrane Library, CNKI, Wanfang, Weipu, CBM, Clinicaltrials.gov and gray literature) were retrieved as of 1^st^ August, 2024. The standardized mean difference (SMD) and 95% confidence interval (CI) represent pooled effect size. We used the Newcastle–Ottawa Scale to evaluate the quality of articles and the certainty of evidence assessed by GRADE system. All statistical analyses were performed using RevMan 5.3 and Stata 12(Stata Corporation, yi TX).

**Results:**

Fifteen case-control studies were included. Circulating irisin levels in the MAFLD group were markedly lower than those in the healthy group (SMD=-1.04 [-1.93, -0.14]). Subgroup analyses by race, age, severity and T2DM revealed that circulating irisin levels were lower in the MAFLD group compared to those in the healthy controls in the Asian population (SMD=-1.38 [-2.44, -0.31], P<0.05) and in those above 50 years old (SMD=-2.23 [-3.64, -0.81], P<0.05) and higher in the mild MAFLD groups than those in moderate to severe MAFLD groups (SMD = 11.68 [9.05, 14.31], P<0.05). And the circulating irisin levels in MAFLD patients with T2DM were significantly lower than those in healthy group (SMD = -2.90 [-4.49, -1.30]). ELISA kits from different companies also presented different relationships.

**Conclusions:**

There were significantly lower circulating irisin levels in the MAFLD group than in the healthy control group. Although these results differed from our previous results, there is no denying that circulating irisin levels are closely associated with the advancement of MAFLD.

## Introduction

Recently, non-alcoholic fatty liver disease (NAFLD) has emerged as the most prevalent chronic liver disease, affecting nearly a quarter of the worldwide population (1). NAFLD is intensely affiliated with metabolic syndromes and was renamed metabolic dysfunction-associated fatty liver disease (MAFLD) in 2020 (2). This innovative designation focuses on the presence of metabolic comorbidities and steatosis (3). MAFLD can progress from relatively benign isolated hepatic steatosis (HS) to more severe non-alcoholic steatohepatitis (NASH), liver fibrosis, cirrhosis, and hepatocellular carcinoma (HCC). With changes in people’s lifestyle and dietary habits, the incidence of MAFLD has increased rapidly year after year (4). The average age of onset is also decreasing, and the prevalence of MAFLD in people under 60 years of age has gradually increased (by 17.8% from 2007 to 2010 and by 46.5% from 2015 to 2018) more than it has in people over 60 years of age (by 23.9% from 2007 to 2010 and by 30.9% from 2015 to 2018) (5). Currently, liver biopsy is still the gold standard for the diagnosis of NAFLD, but due to its invasiveness, sampling variability, high cost, and other limitations, its acceptance is low (6). The accuracy of imaging tests needs to be improved, and because the diagnostic ability of many molecular markers is also limited, more accurate biomarkers are needed to effectively predict MAFLD disease and its progression.

Irisin is a hormone-like myokine that is released by cleavage of the ectodomain of the transmembrane receptor fibronectin type III domain-containing 5 (FNDC5), which is conveyed in skeletal muscle and other tissues (7). Since the publication of Bostrom et al. (8) on irisin in 2012, it has rapidly become a research hotspot and has been related to a series of metabolic disorders and various liver diseases. Irisin can increase energy consumption by stimulating “browning” of white adipose tissue, participates in energy metabolism, and improves glucose homeostasis by lowering insulin resistance (9). In addition, irisin can also serve as an adipokine that regulates the metabolic rate of human adipose tissue (10). Recently, Armandi et al. (11) found that circulating irisin levels may serve as a potential biomarker for predicting NAFLD severity. In addition, other scientific studies have shown that circulating irisin levels may be negatively correlated with obesity, may also be associated with fat degeneration, and may decrease with liver damage (12). Furthermore, Zhu W. et al. (13) explained the important regulatory role of irisin in NAFLD at the mechanistic level using a high-fat diet (HFD)-induced NAFLD mouse model. Irisin is inextricably linked to the development of MAFLD, and its expression level may reflect the degree of disease.

Numerous scholars have explored the circulating irisin levels in MAFLD patients, but the results have been conflicting. We also performed a meta-analysis in 2020. However, because of the limited number of incorporated studies, the inadequate sample size of some studies, and the bias of selection of the study population, we updated the previous studies, increased the number of studies, and further detailed the inclusion criteria, hoping to obtain more comprehensive and accurate conclusions to facilitate clinical and scientific research, better assess the relationship between MAFLD and circulating irisin levels, and explore potential biomarkers and novel treatment targets for MAFLD.

## 2 Methods

### 2.1 Data sources and search strategy

**Figure 1:**
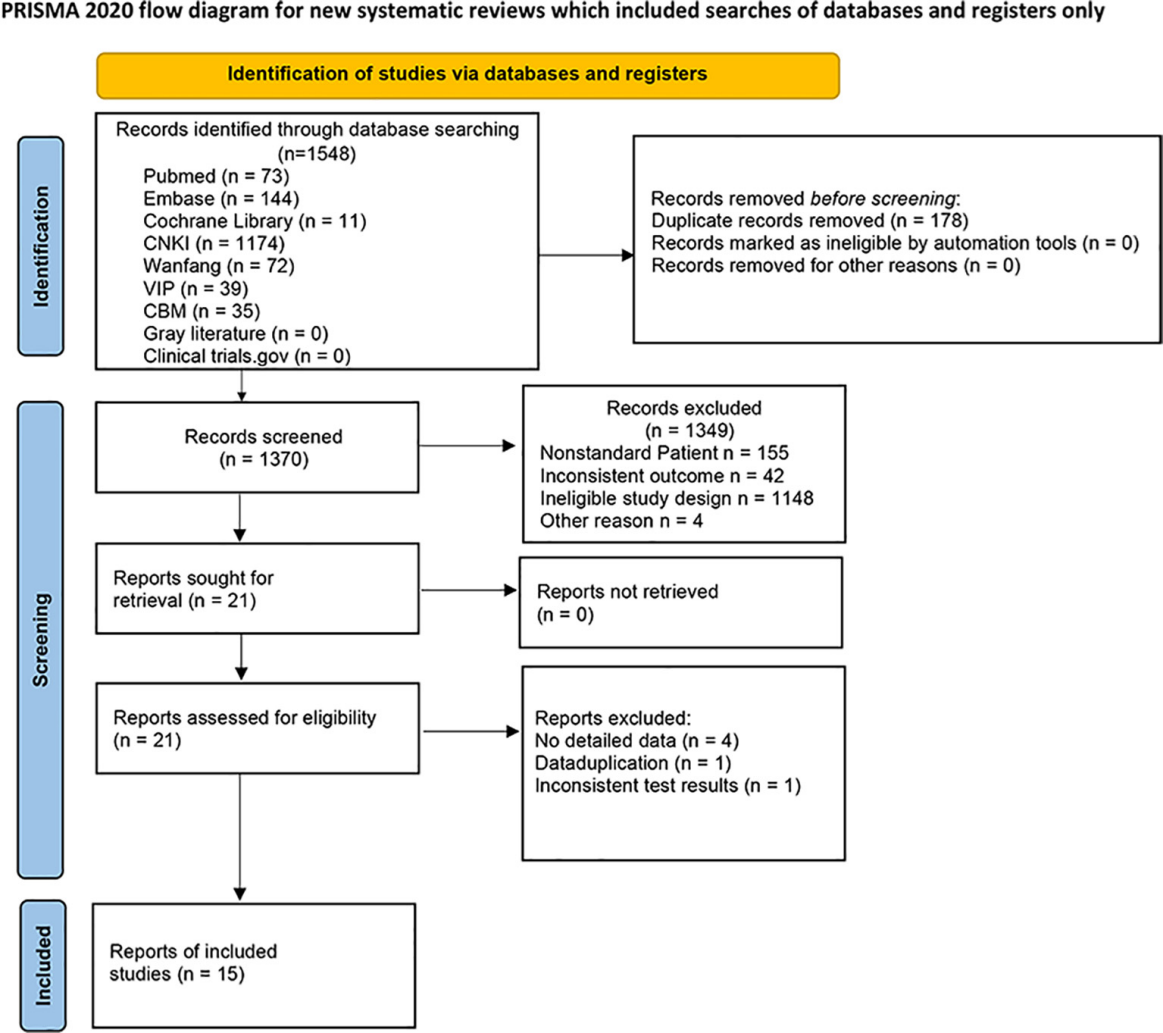
Flowchart of study inclusions and exclusions.

The study was proceeded in accordance with Cochrane Collaboration guidelines and reported based on the Preferred Reporting Items for Systematic Reviews and Meta-Analyses (PRISMA) criteria (**Figure 1**). The research protocol was registered in PROSPERO (number CRD42019130962) (**Supplementary Material 1**).

All articles originated from nine databases (PubMed, EMBASE, Cochrane Library, CNKI, Wanfang, Weipu, CBM, Clinicaltrials.gov, grey literature) until August 01, 2024. The following free and MeSH search terms were used: (“Non-alcoholic Fatty Liver Disease”, “NASH”, “Nonalcoholic fatty liver disease”, “Non alcoholic fatty liver disease”, “Non alcoholic Fatty Liver Disease”, “NAFLD”, “Nonalcoholic Fatty Liver Disease”, “Nonalcoholic Fatty Liver”, “Nonalcoholic Steatohepatitis”, “Nonalcoholic Steatohepatitides”, “fatty liver”, “Liver, Nonalcoholic Fatty”, “Steatohepatitides, Nonalcoholic”, “Steatohepatitis, Nonalcoholic”, “Metabolic Associated Fatty Liver Disease”, “MAFLD”, “Metabolic dysfunction-associated fatty liver disease”, “Metabolic associated steatohepatitis”, “MASH”, “Metabolically Associated Liver Steatosis”) and (“irisin”, “fibronectin type III domain containing protein 5”, “Fndc5 protein”, “FNDC5”, “FRCP2 protein”), The complete search strategy is fully elaborated in **Supplementary Material 2**. In addition, to ensure no omissions, relevant citations in the literature were searched. For documents with incomplete content, the authors were contacted via e-mail for more detailed data. No language restrictions were applied.

### 2.2 Inclusion and exclusion criteria

Each piece of literature was reviewed by two researchers individually, and the controversial parts were handed over to a third member for arbitration based on our protocol.

The inclusion criteria of studies were (1) patients aged 18 years or older with MAFLD, NAFLD, simple fatty liver (SFL), nonalcoholic fatty liver (NAFL) or NASH; (2) MAFLD diagnosed in accordance with significant hepatocellular steatosis on liver biopsy histology or diffuse fatty liver disease on imaging; (3) control group comprising of healthy individuals who don’t have any metabolic diseases; (4) circulating irisin levels from serum or plasma and (5) a case-control or cohort study design. The case group included patients with NAFLD or MAFLD, and the control group consisted of healthy individuals without NAFLD in case-control studies. In cohort studies, the groups were divided into high irisin level and low irisin level groups based on circulating irisin levels.

The exclusion criteria were detailed below: (1) studies including secondary liver fat accumulation caused by heavy alcohol drinking and other well-defined liver-damaging factors (such as hereditary factors, viruses, and drugs); (2) no comparison with healthy individuals; (3) full text that does not focus on circulating irisin levels (serum or plasma); (4) clinical randomized controlled trials, case reports, animal experimental studies, or literature review; (5) repetitive publications; (6) articles with insufficient data and obstruction in data retrieval due to corresponding authors who are unresponsive.

### 2.3 Data extraction

Two investigators separately fetched information which included the first author’s name, publication year, country, Newcastle–Ottawa Scale (NOS) score, diagnosis method of the disease, the number and basic information of the case and control groups (including age and body mass index [BMI]), diagnosis standards of MAFLD, circulating levels of irisin in the case and control groups, and Homeostasis model assessment of insulin resistance (HOMA-IR) levels of the case and control groups.

### 2.4 Quality evaluation and risk of bias

The included studies were evaluated employing the NOS (14), which consists of selection, comparability, and exposure. The Grades of Recommendation, Assessment, Development, and Evaluation (GRADE) approach provides guidance for assessing the quality of the study (https://gdt.gradepro.org). For publication bias, the Egger’s test (15) was used. Quality appraisal was carried out autonomously by two researchers, and the results would be validated by a third researcher if there was any divergence.

### 2.5 Statistical analysis

Revman 5.3 and Stata 12 (Stata Corporation, College Station, TX, USA) were utilized for statistics analyses. Normally distributed data were extracted as mean ± Standard Deviation (SD), and those with a non-normal distribution were transformed to the mean ± SD (https://www.math.hkbu.edu.hk/~tongt/papers/median2mean.html) (16–18).

We utilized the standardized mean difference (SMD) to extract all circulating levels of irisin as continuous variables. The heterogeneity level was estimated on basis of the P-value and I^2^ test. When there was observed significant statistical heterogeneity (I^2^ ≥ 50%), the random effects model (shown as “D+L”) was recommended (19, 20). In the absence of significant statistical heterogeneity (I^2^ < 50%), the fixed effects model (shown as “I-V”) was adopted. Subgroup analysis and meta-regression were applied to identify the fundamental causes of high heterogeneity. Subgroup analysis were executed from several perspectives, including race, BMI, age, HOMA-IR, NOS score, severity, ELISA kits and presence or absence of T2DM. As BMI ≥25 kg/m^2^ was considered overweight, patients were classified into two subgroups in accordance with BMI≥25 and BMI <25 kg/m^2^ (21). For univariate meta-regression results showing P < 0.1 (22), it is meaningful to explain the partial heterogeneity of our study. Sensitivity analysis was conducted by omitting each study at a time.

## 3 Results

### 3.1 Study selection

The initial search yielded 1548 articles. After removing duplicates and screening titles, abstracts, and entire texts according to the inclusion and exclusion criteria, 15 studies were included in the data analysis (23–37). The detailed literature screening flow is illustrated in **Figure 1**. Fifteen studies were included in the meta-analysis. Eight studies were conducted in China (23–30), two in Greece (33, 34), one in South Korea (31), one in Iran (36), one in Poland (37), one in Japan (32), and one in Egypt (35). All the studies followed a case-control study design. The patient and control groups were not restricted by sex, and the average age ranged from 33 to 68 years. The BMI of patients with MAFLD ranged from 20.3 to 35.91 kg/m^2^, whereas that of healthy controls had a range of 20.5 to 30.9 kg/m^2^ (details are shown in **Table 1**).

**Table 1:** Baseline characteristics of studies included in the meta-analysis.

n	First authors	Year	Country		No. of Patients		Sex(M/W)		Age		BMI (kg/m^2^)		Detection Method
					MAFLD group	Control group	MAFLD group	Control group	MAFLD group	Control group	MAFLD group	Control group	
1	Choi (32)	2014	South Korea	-	84	271	38/46	56/215	48.00 ± 9.80	44.40 ± 9.90	25.70 ± 3.00	22.40 ± 2.70	ELISA kit (R&D, Minneapolis, MN, USA)
2	Guo (23)	2020	China	-	120	120	59/61	61/59	41.72 ± 8.29	40.48 ± 8.22	26.51 ± 1.72	24.71 ± 2.14	ELISA
3	Liu (24)	2018	China	T2DM+NAFLD	102	60	56/46	32/28	56.32 ± 9.36	56.18 ± 9.20	26.48 ± 4.68	23.12 ± 3.54	ELISA
4	Lou (25)	2017	China	-	140	148	97/43	101/47	48.69 ± 8.63	47.36 ± 8.43	22.01 ± 2.28	21.36 ± 2.46	/
5	Polyzos (33)	2013	Greece	NAFLD	15	Lean 24	5/10	4/20	53.90 ± 2.60	54.20 ± 1.60	31.90 ± 1.30	25.30 ± 0.30	ELISA kit (Phoenix Pharmaceuticals, CA, USA)
				NASH	16	Obese 28	3/13	8/20	53.90 ± 2.90	52.60 ± 1.60	34.10 ± 1.40	30.90 ± 0.60	
6	Polyzos (34)	2014	Greece	-	30	26	8/22	6/20	53.80 ± 2.00	53.00 ± 1.80	33.00 ± 1.00	30.30 ± 0.70	ELISA kit (Phoenix Pharmaceuticals, CA, USA)
7	Ren (26)	2017	China	T2DM+mild NAFLD	40	40	23/17	22/18	49.00 ± 6.00	49.00 ± 6.00	24.30 ± 2.30	22.30 ± 2.60	ELISA
				T2DM+moderate to severe NAFLD	40	40	21/19	22/18	50.00 ± 7.00	49.00 ± 6.00	28.40 ± 3.10	22.30 ± 2.60	
8	Rizk (35)	2015	Egypt	-	20	20	9/11	10/10	47.85 ± 6.24	44.25 ± 10.46	35.91 ± 3.00	35.91 ± 3.00	ELISA kit (BioVendor, Bmo, CZ)
9	Shanaki (36)	2017	Iran	NAFLD	41	40	40/0	40/0	51.60 ± 0.90	52.40 ± 1.30	29.00 ± 0.49	24.04 ± 0.73	ELISA kit (BioVendor, Brno, CZ)
				T2DM+NAFLD	40	40	40/0	40/0	53.90 ± 1.20	52.40 ± 1.30	29.25 ± 0.62	24.04 ± 0.73	
10	So (31)	2014	Japan	-	274	37	/	/	52.00 ± 12.00	28.00 ± 10.00	/	/	ELISA
11	Waluga (37)	2019	Poland	-	25	25	12/13	11/14	31.00 ± 10.00	42.00 ± 15.00	31.14 ± 6.07	22.15 ± 0.83	ELISA kit (BioVendor, Brno, CZ)
12	Wu (27)	2015	China	-	135	150	90/45	103/47	47.87 ± 9.12	48.25 ± 8.16	26.10 ± 2.40	22.40 ± 2.70	ELISA kit (R&D, Minneapolis, MN, USA)
13	Xu (28)	2019	China	-	60	60	46/14	42/18	46.00 ± 8.00	45.00 ± 11.00	24.80 ± 2.20	22.00 ± 2.40	ELISA kit (Aviscera Bio sciences, Santa Clara, CA, USA)
14	Zhang (30)	2013	China	-	650	393	215/435	83/310	54.00 ± 7.20	52.70 ± 7.30	28.00 ± 2.70	26.40 ± 2.20	ELISA kit (Aviscera Bio sciences, Santa Clara, CA, USA)
15	Zhang (29)	2021	China	T2DM+NAFLD	88	90	48/40	53/37	59.33 ± 8.69	60.11 ± 8.82	20.30 ± 3.20	20.50 ± 3.10	ELISA kit (Elabscience, Wuhan, China)

Ranked by beginning letter of the first author; Abbreviations: M, Man; W, Woman; BMI, Body mass index; MAFLD, metabolic associated fatty liver disease; ELISA, enzyme-linked immune-sorbent assay; T2DM, Type 2 diabetes mellitus.

### 3.2 Quality assessment

The average NOS score for all included studies was 6.4, indicating that most of the studies were of high quality. Except for the studies by So et al. (32), Lou et al. (25), Zhang et al. (30), Waluga et al. (37), and Polyzos et al. (2014) (33), the other 10 studies all scored 7 or higher. The representativeness of cases and the selection or definition of controls were not adequately described, which is a problem for the three articles with scores below 7 (**Table 2**). The GRADE approach results indicated that the general quality of evidence was exceedingly low (shown in **Supplementary Material 3**). Observational studies were scored low. Significant heterogeneity among the included studies led to serious inconsistencies (I^2^ > 90%). In addition, the basic information of the case and control groups in some articles was not strictly matched, which also has an impact on the determination of the evidence.

**Table 2:** Newcastle-Ottawa Scale (NOS) score of included articles.

No.	Author	Year	Selection				Comparability	Exposure			Total	Average
			Adequate definition	Representativeness	Selection of controls	Definition of controls		Ascertainment of exposure	Same method	Non-response rate		
1	Choi (32)	2014	1	1	1	1	0	1	1	1	7	
2	Guo (23)	2020	1	1	1	1	1	1	1	1	8	
3	Liu (24)	2018	1	1	1	1	0	1	1	1	7	
4	Lou (25)	2017	1	1	1	1	0	0	1	1	6	
5	Polyzos (33)	2013	1	1	1	1	0	1	1	1	7	
6	Polyzos (34)	2014	1	0	0	0	0	1	1	1	4	
7	Ren (26)	2017	1	1	1	1	0	1	1	1	7	
8	Rizk (35)	2015	1	1	1	1	0	1	1	1	7	6.4
9	Shanaki (36)	2017	1	1	1	1	0	1	1	1	7	
10	So (31)	2014	0	0	0	1	0	1	1	1	4	
11	Waluga (37)	2019	1	0	0	1	0	1	1	1	5	
12	Wu (27)	2015	1	1	1	1	0	1	1	1	7	
13	Xu (28)	2019	1	1	1	1	0	2	1	1	7	
14	Zhang (30)	2013	1	0	1	1	0	1	1	1	6	
15	Zhang (29)	2021	1	1	1	1	0	1	1	1	7	

### 3.3 Association between circulating irisin levels and MAFLD

**Figure 2:**
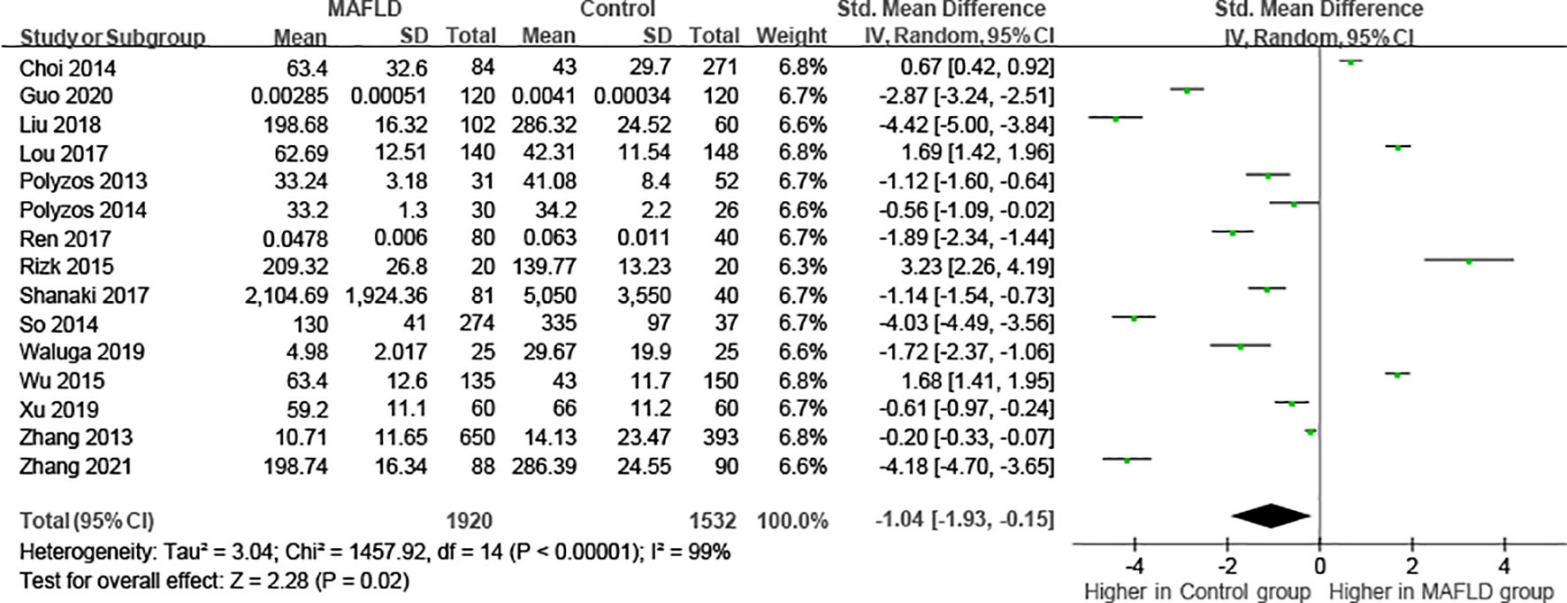
Forest plot of circulating irisin levels between MAFLD and the healthy control group (Random-Effects Model, SMD).

**Figure 3:**
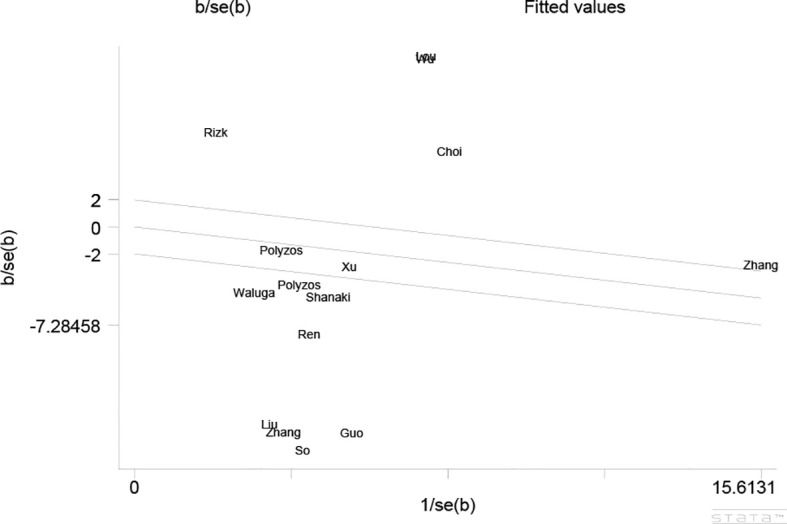
Galbraith Test result of circulating irisin levels between MAFLD and the healthy control group.

As the results of the pooled meta-analysis (**Figure 2**) showed significant heterogeneity (I^2^ = 99%, P < 0.001), a random-effects model was used. The results demonstrated that in the MAFLD group had significantly lower circulating irisin level than the healthy group, with an overall SMD (95% confidence interval [CI]) of -1.04 (-1.93, -0.14) (**Figure 2**). Simultaneously, we performed heterogeneity analyses using the Galbraith test, which suggested that fewer than 50% of the studies were within an acceptable limit (**Figure 3**). Consequently, it was necessary to further explore potential reasons of heterogeneity through subgroup analysis and meta-regression.

### 3.4 Subgroup analysis

The results of the subgroup analysis are shown in **Table 3**, with high heterogeneity accounting for the use of the random-effects model. Subgroup analyses were performed in light of race, BMI, age, HOMA-IR, NOS, disease severity, different ELISA kits and presence or absence of T2DM (forest plot of subgroups is shown in **Supplementary Material 4**). Most subgroup results showed no prominent variation in circulating irisin levels comparing the MAFLD group to healthy controls. However, it is important to note that the findings from subgroup analysis stratified by race indicated that in Asian populations, circulating irisin levels were lower in the MAFLD group than in healthy controls, with an overall SMD (95% CI) of -1.38 (-2.44, -0.31). Subgroup analysis results by age showed that in patients aged >50 years, circulating irisin levels in the MAFLD group were lower than those in the healthy group (SMD = -2.23 [-3.64, -0.81]), and subgroup analyses classified by severity revealed that higher circulating irisin levels were found in both mild and moderate to severe MAFLD groups than in the healthy group (mild: SMD =1.69 [0.80, 2.57]; moderate to severe: SMD = 1.03 [0.77, 1.29]). In addition, we also compared mild MAFLD with moderate to severe MAFLD and showed that the circulating irisin levels in the mild MAFLD group were observed higher as compared to those in the moderate to severe MAFLD group (SMD = 11.68 [9.05, 14.31]). Besides, we also performed a subgroup analysis based on inclusion or exclusion of T2DM, and the results showed that the circulating irisin levels in MAFLD patients with T2DM were significantly lower than those in healthy group (SMD = -2.90 [-4.49, -1.30]). Moreover, ELISA kits had significant influence on the results. Irisin detected with ELISA kits (Aviscera Bio sciences, Santa Clara, CA, USA; BioVendor, Bmo, CZ; Unspecified) were irrelevant (SMD = -0.37 [-0.76, 0.03]; SMD = 0.08 [-2.21, 2.38]; SMD = -2.30 [-4.82, 0.22]), ELISA kits produced by R&D, Minneapolis, MN, USA was positively correlated (SMD = 1.17 [0.18, 2.16]), whereas the ELISA kits (Phoenix Pharmaceuticals, CA, USA; Elabscience, Wuhan, China) are lower than in healthy people (SMD = -0.85 [-1.40, -0.30]; SMD = -4.18 [-4.70, -3.65]). However, no clear source of heterogeneity was identified in subgroup analyses.

**Table 3:** Subgroup analysis of the circulating irisin levels in MAFLD patients compared with controls.

Subgroups	Classification	Studies	SMD (95%CI)	I^2^(%)	P for heterogeneity
race	Asian	11	-1.38[-2.44, -0.31]	99	P<0.00001
	Others	4	-0.09[-1.64,1.46]	96	P<0.00001
	Total(95%CI)	15	-1.04[-1.93, -0.15]	99	P<0.00001
BMI	BMI>25	11	-0.77[-1.68,0.13]	99	P<0.00001
	BMI ≤ 25	3	-1.02[-4.07,2.02]	99	P<0.00001
	Total(95%CI)	14	-0.82[-1.68,0.04]	99	P<0.00001
age	age>50	7	-2.23[-3.64, -0.81]	99	P<0.00001
	age ≤ 50	8	0.01[-1.25,1.26]	99	P<0.00001
	Total(95%CI)	15	-1.04[-1.93, -0.15]	99	P<0.00001
HOMA-IR	HOMA-IR>5	6	-0.52[-2.12,1.09]	99	P<0.00001
	HOMA-IR ≤ 5	6	-0.03[-0.76,0.69]	96	P<0.00001
	Total(95%CI)	12	-0.25[-1.03,0.52]	99	P<0.00001
NOS score	NOS score>5	12	-0.77[-1.72,0.18]	99	P<0.00001
	NOS score ≤ 5	3	-2.10[-4.29,0.08]	98	P<0.00001
	Total(95%CI)	15	-1.04[-1.93, -0.15]	99	P<0.00001
severity	mild	3	1.69[0.80,2.57]	95	P<0.00001
	moderate to severe	3	1.03[0.77,1.29]	56	P=0.03
	Total(95%CI)	3	1.24[0.86,1.63]	89	P<0.00001
T2DM	With T2DM	4	-2.90 [-4.49, -1.30]	98	P<0.00001
	others	11	-0.36 [-1.29, 0.57]	99	P<0.00001
	Total(95%CI)	15	-1.04 [-1.93, -0.15]	99	P<0.00001
ELISA kit	Aviscera Bio sciences, Santa Clara, CA, USA	2	-0.37 [-0.76, 0.03]	76	P = 0.04
	R&D, Minneapolis, MN, USA	2	1.17 [0.18, 2.16]	97	P < 0.00001
	Phoenix Pharmaceuticals, CA, USA	2	-0.85 [-1.40, -0.30]	58	P = 0.12
	Elabscience, Wuhan, China	1	-4.18 [-4.70, -3.65]	/	P < 0.00001
	BioVendor, Bmo, CZ	3	0.08 [-2.21, 2.38]	97	P < 0.00001
	Unspecified	5	-2.30 [-4.82, 0.22]	99	P < 0.00001
	Total(95%CI)	15	-1.04 [-1.93, -0.15]	99	P < 0.00001

BMI, body mass index; SMD, standardized mean difference; NOS, Newcastle-Ottawa Quality Assessment Scale; HOMA-IR, Homeostasis model assessment of insulin resistance; T2DM, Type 2 Diabetes Mellitus; ELISA, enzyme-linked immune-sorbent assay; CI, Confidence interval.

### 3.5 Meta-regression

The sources of heterogeneity were further explored using a meta-regression. Univariate regression analysis showed that the group classified based on the presence of T2DM has a P-value <0.05, which can be interpreted as a source of heterogeneity. Age and severity P-values were <0.1. Race, BMI, HOMA-IR, NOS score, Fasting Blood Glucose (FBG), and alanine transaminase (ALT) and aspartate transaminase (AST) levels may not account for the origins of heterogeneity (race, P = 0.338; BMI: P = 0.865; HOMA-IR: P = 0.865; FBG: P = 0.305; ALT: P = 0.418; AST: P = 0.734). Multivariate meta-regression was not performed because of the insufficient number of studies available for analysis and too few studies in which the two factors overlapped. Due to the paucity of studies, univariate regression of the ELISA kits was not feasible. The results of the meta-regression analysis are presented in **Table 4** (see **Supplementary Material 5** for details).

**Table 4:** meta-regression of MAFLD and circulating irisin levels.

Covariates	No. Studies	Coefficient	Standard error	*t*	*P*	95%CI
race	14	1.33	1.33	1.00	0.338	[-1.55,4.20]
BMI	14	-0.26	1.48	-0.17	0.86	[-3.49,2.98]
age	15	2.25	1.05	2.15	0.051	[-.014,4.51]
HOMA-IR	12	0.55	1.05	0.52	0.615	[-1.80,2.89]
NOS Score	15	-1.35	1.48	-0.91	0.378	[-4.54,1.84]
FBG	14	-1.26	1.18	-1.07	0.305	[-3.83,1.31]
ALT	12	-1.04	1.23	-0.84	0.418	[-3.78,1.70]
AST	12	-0.51	1.47	-0.35	0.734	[-3.79,2.76]
severity	10	-0.65	0.33	-1.94	0.089	[-1.42,0.12]
T2DM	15	2.56	1.18	2.17	0.049	[-10.03, -.91]

CI, Confidence interval; BMI, Body mass index; HOMA-IR, Homeostasis model assessment of insulin resistance; NOS, Newcastle-Ottawa Scale; FBG, Fasting Blood Glucose; ALT, alanine aminotransferase; AST, aspartate aminotransferase; T2DM, Type 2 Diabetes Mellitus.

### 3.6 Sensitivity analysis

**Figure 4:**
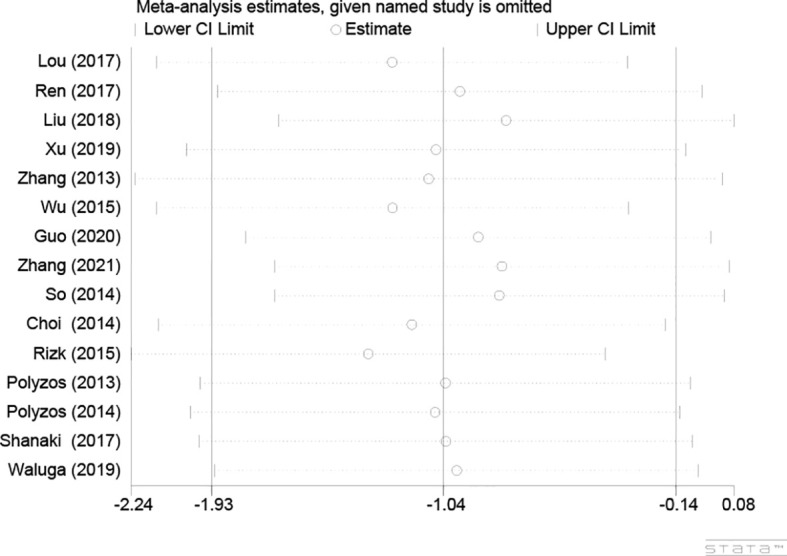
Sensitivity analysis plot of circulating irisin levels between MAFLD and the healthy control group.

One-by-one removal of the included studies was used for sensitivity analysis to observe changes in the pooled effects. As shown in **Figure 4**, the result was not influenced by any of the studies, confirming the stability of this study.

### 3.7 Publication bias

**Figure 5:**
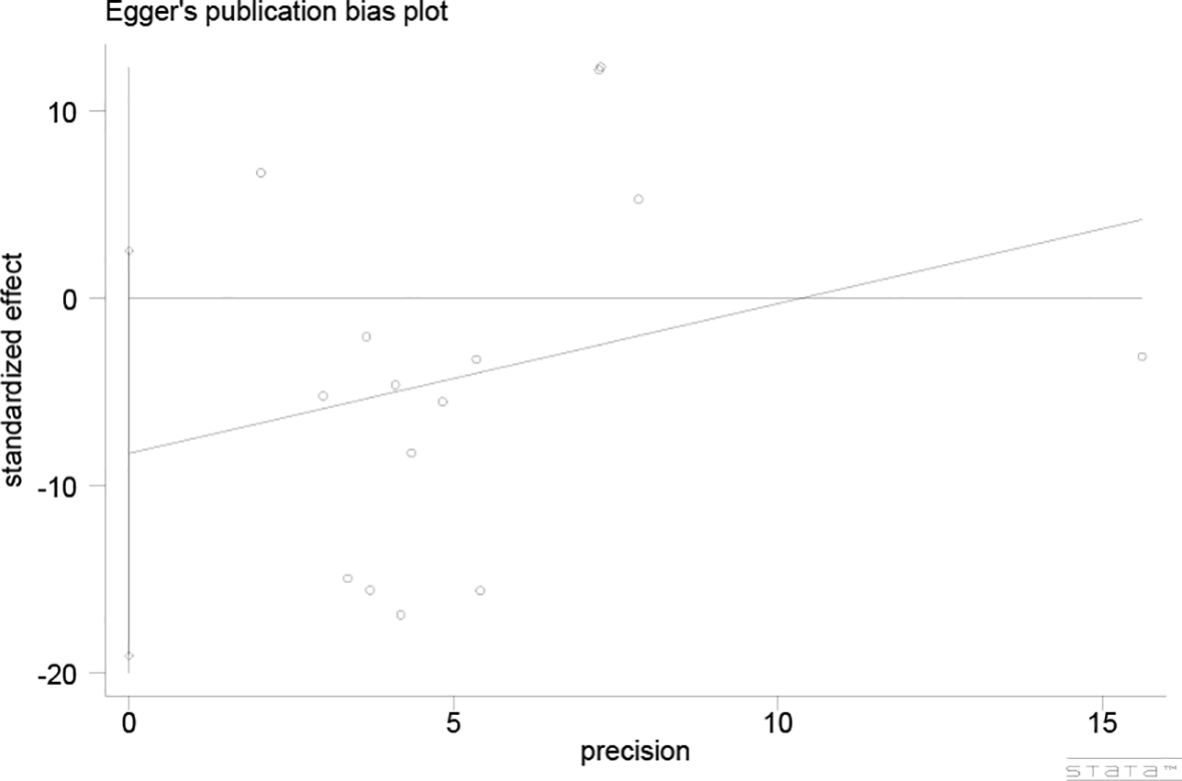
Egger’s publication bias plot.

The Egger’s test was utilized to assess publication bias (**Figure 5**), which demonstrated a low potential for publication bias (P = 0.122, P > 0.05).

## 4 Discussion

This current meta-analysis aimed to explore the connection between circulating irisin level and MAFLD. Currently, MAFLD is considered the most common chronic liver disease. MAFLD is considered a more appropriate general term than NAFLD (38). Many studies have suggested that overweight or obesity (39), type 2 diabetes mellitus (T2DM), and metabolic dysfunction (40, 41) are closely related to the complicated mechanism underlying the prevalence and development of MAFLD (2).

Irisin, a well-defined factor secreted by muscles, regulates metabolism and prevents obesity, which is associated with the occurrence and progression of MAFLD. Irisin can activate liver autophagy, reduce liver inflammation, and promote the browning of white fat cells (42, 43) through sports activities (44, 45), thus improving MAFLD. Polyzos et al. (33) showed that serum irisin levels were lower in obese controls and in patients with NAFLD and NASH than those in lean controls. Zhang et al. (46) also indicated that serum levels of irisin were reduced in NAFLD- afflicted obese adults. Ulualan et al. (12) found that serum irisin levels were lower in obese patients with and without NAFLD than in a control group. Gonzales-Gil et al. (47) reported that patients with obesity and metabolic syndrome (MS) had significantly lower plasma irisin levels compared to normal-weight controls. In addition, Choi et al. (31) reported that serum irisin levels were lower in patients with severe steatosis than in those with mild steatosis. Related research on irisin and MAFLD is constantly updated. Therefore, the latest systematic review and meta-analysis of the relationship between irisin and MAFLD was implemented for early clinical diagnosis and further scientific exploration.

In this study, 1548 articles were retrieved from both English and Chinese databases (PubMed, EMBASE, Cochrane Library, CNKI, Wanfang, CBM, VIP, Clinicaltrials.gov and gray literature), and 15 studies were ultimately enrolled, containing eight studies from China, two from Greece, one from South Korea, one from Iran, one from Poland, one from Japan, and one from Egypt. The circulating irisin level in patients with MAFLD was significantly lower than that in healthy controls. Patients with MAFLD have worse liver impairment than healthy people, and previous studies have found that circulating irisin levels decrease with liver damage (31, 36, 46). Meanwhile, irisin is produced during physical exercise (8, 48, 49) and decreases after long periods of inactivity. However, many diseases such as obesity, T2DM, and metabolic dysfunction are caused by a sedentary lifestyle, which may not be conducive to circulating irisin (50). High heterogeneity cannot be ignored; therefore, subgroup analysis and meta-regression were crucial to find the potential root causes of heterogeneity. From the perspective of race, the circulating irisin level was lower in the MAFLD group than that in healthy controls in Asian populations, but there was no significant difference in other subgroups. Subgroup analysis based on severity observed that both mild and moderate to severe MAFLD groups had higher circulating irisin levels than healthy controls, likewise mild MAFLD groups had higher irisin levels than moderate to severe MAFLD groups. It was indicated that the higher severity of MAFLD was connected with the lower irisin levels, which was not in conflict with our general results and conclusions. In terms of combined with T2DM, obvious lower circulating irisin levels were shown in MAFLD patients with T2DM compared to those in healthy controls. However, when BMI, age, HOMA-IR, and NOS score were used for subgroup analysis, it could not perceive significant difference between the MAFLD and healthy groups. Based on univariate meta-regression results, age and severity may be the sources of heterogeneity. However, as there was no common study comparing age and severity, multivariate meta-regression could not be conducted. Sensitivity and publication bias analyses were conducted. Excluding any study did not significantly influence the results. In addition, the Egger’s test demonstrated a low possibility of publication bias. These results indicate that our study is persuasive and representative to some extent.

Fifteen articles were included in this study, consisting of 1920 patients and 1532 healthy controls, which improved the validity of publication bias over previously published studies. Several studies, including one by as Shanaki et al. (36), on MAFLD patients with overweight, obesity, T2DM, and metabolic dysfunction were also included based on the updated definition of MAFLD. However, many studies were excluded for various reasons. For example, Zhang et al. published two studies (30, 46), and since the data were the same, we included a more detailed one (30). A study by Metwally et al. (51) had no controls, which did not conform to our study design criteria. The control group in Canivet et al. (52) included lean subjects and non-healthy controls, which was inconsistent with the study design criteria and was therefore also excluded. Clinicaltrials.gov and the gray literature were also scrutinizeed to resolve publication bias, which was shown to be low according to the Egger’s test (P = 0.122, P > 0.05). Regarding the use of different ELISA kits to measure irisin levels, the SMD of irisin was chosen as the primary result rather than the weighted mean difference (WMD), so variations in the different reference ranges of different ELISA kits were adjusted based on their associated SDs. Different ELISA kits significantly affect the results. Circlulating irisin levels detected with ELISA kits [Aviscera Bio sciences, Santa Clara, CA, USA (28, 30); BioVendor, Bmo, CZ (35–37); Unspecified (23–26, 31)] were irrelevant. There were higher circlulating irisin levels obtained by R&D, Minneapolis, MN, USA (27, 32) in MAFLD individuals compared to healthy group. Serum irisin levels in the MAFLD group were less than those in the healthy control group when ELISA kits produced by Pharmaceuticals, CA, USA (33, 34) and Elabscience, Wuhan, China (29). (See **Table 1** for details.)

Recently, Qiu et al. (53) updated a meta-analysis of NAFLD versus circulating irisin levels and found no difference exciting in the level of circulating irisin between NAFLD and non-NAFLD patients. First, in terms of the literature search, Qiu et al. (53) mainly searched English databases, and our team added a search of Chinese databases to make the included studies more comprehensive. Second, there were also differences in literature inclusion, and the control population included by Qiu et al. (53) was more extensive than the non-NAFLD control group or patients in different disease stages of NAFLD. We developed screening criteria in greater detail based on the latest MAFLD guidelines, with a greater focus on rigorous screening of the included population. Among the studies included by Qiu et al. (53), five studies were not included by us, namely Canivet et al. (52), Metwally et al. (51), Monserrat-Mesquida et al. (54), Moreno-Perez et al. (55), and Petta et al. (56) The reasons for the exclusion of Canivet et al. (52) and Metwally et al. (51) are mentioned above. The study by Monserrat-Mesquida et al. (54) did not explicitly mention that the included population was NAFLD patients, and Moreno-Perez et al. (55) included HIV patients, which did not meet our inclusion criteria. Petta et al. (56) included patients with compensated cirrhosis caused by NASH, whereas our criteria excluded patients with NAFLD-associated cirrhosis. More importantly, the results of Qiu et al. (53) were inconsistent with those of our group, as we found that the circulating irisin level in patients with MAFLD was lower than that in healthy controls.

Similarly, our updated meta-analysis also differed from previous conclusions, and differences in the included articles may be the main reason. In addition, contrary to our previous study, this meta-analysis suggests that the MAFLD group might exhibit significantly reduced circulating irisin levels than the healthy group. However, previous study indicated that the circulating irisin levels were not significantly different between the NAFLD group and healthy controls. First of all, with regard to literature retrieval, the updated study retrieved more than 1,000 additional literatures. Secondly, there were differences in literature inclusion, the updated paper incorporated more extensive articles than previous one. Studies on NAFLD combined with obesity or T2DM were not excluded in this updated article due to the MAFLD standard guidelines and the inclusion criteria adjustments. Meanwhile, the updated research demonstrated that the circulating irisin levels in MAFLD patients with T2DM were significantly lower than those in healthy group (24, 26, 29, 36). So it can be deduced that the insufficient number of included articles and analysis samples in our original study might affect the accuracy of previous research results.

Besides, most observational studies have shown that MAFLD patients present lower circulating irisin levels than healthy individuals. Aydin et al. (57) found through animal experiments that the circulating irisin level in rats with liver damage was reduced, and if MAFLD patients allow the disease to progress, it may lead to liver cell damage, which also provides a basis for our research results. Du et al. (58) suggested that circulating irisin levels were decreased in patients with T2DM, and relevant studies shown that the prevalence of MAFLD is higher in people with diabetes than in people without diabetes (59, 60).

Therefore, the updated discovery could not only help to diagnose the disease earlier but also develop the diets of patients. But there is relatively few food related literature about improving the circulating irisin levels. Fortunately, because irisin is produced primarily by exercise-induced muscle secretion and is associated with vitamin D, several studies have provided dietary information indirectly. For example, high-protein foods (8, 61, 62) and high-vitamin foods (63–66) like milk, fish, meat, vegetables, fruits, can help increase the circulating irisin levels, which can provide more precious and beneficial dietary guidance for MAFLD patients. More and higher-quality researches are needed to determine and foster daily dietary guidelines for MAFLD individuals.

### 4.1 Study strengths and limitations

This meta-analysis was primarily focused on the association between circulating irisin levels and MAFLD. To avoid local literature bias, studies in both English and Chinese languages were included, and Clinicaltrials.gov and gray literature were also searched to reduce publication bias. Subgroup univariate regression analysis was used to study the data, and the results were interpreted and discussed. In addition, the stability of the merged results was affirmed by the Egger’s test and sensitivity analysis. However, this meta-analysis had several limitations that cannot be ignored. First of all, only 15 articles were included in this updated study, the number of articles in this study was insufficient and not entirely representative. In addition, more than 70% of the research population in the articles was from Asia. Second, as no study was concerned with both age and severity, multivariate meta-regression could not be performed. If more comprehensive and systematic studies are available, more convincing results can be obtained. Finally, circulating irisin levels in some studies [e.g., Liu et al. (24), Guo et al. (23), and Shanaki et al. (36)] still differed greatly from those in others after unit homogenization, and different ELISA kits were utilized to measure circulating irisin levels, and the reliability of commercially available irisin ELISA kits is still debatable (67–70). Therefore, SMD was adopted instead of WMD to eliminate the impact of different ELISA kits resulting in significant differences in serum irisin levels. It is essential to conduct more serviceable analytical studies so as to explore detection methods of ELISA kits (69), patients physical activities (68), and sampling time. Moreover, several studies were not included owing to the lack of data, and no relevant information was available despite contacting the authors by email. Furthermore, different experimental personnel, operations, and equipment may also affect the reliability of the outcomes. All of these conditions influenced the reliability of the final results. Finally, only three studies with small sample sizes from Asia performed subgroup analysis of disease severity classification. In the future, it is necessary to strictly control inclusion and exclusion criteria, retrieve a larger sample size, develop a more rigorous design, and conduct high-quality randomized controlled trials to explore the accurate effect of irisin signaling in MAFLD pathogenesis, which will provide a new method for the intervention and projection of blood irisin levels related to MAFLD.

## 5 Conclusions

Overall, this meta-analysis suggested that circulating irisin levels in patients with MAFLD were lower than in healthy controls. In the Asian population subgroup and in the subgroup of patients aged >50 years, the circulating irisin levels of patients with MAFLD were lower than those of the healthy control group. Within the severity subgroup, patients with mild, moderate, and severe MAFLD had higher circulating irisin levels compared with the healthy controls and the irisin levels in mild MAFLD patients were higher than those in moderate to severe MAFLD patients. The patients of MAFLD combined with T2DM presented apparently lower circulating irisin levels compared with the healthy groups. Subgroup analysis revealed no sources of heterogeneity. Univariate regression analysis revealed that age and severity P-values were less than 0.1, T2DM P-value was lower than 0.05.

Therefore, the relationship between irisin and MAFLD can be summarized as follows: irisin may serve as a key indicator in MAFLD diagnosis, which is universally challenging to diagnose and treat during initial stages. Furthermore, we discovered that irisin could be an indicator of the progression of MAFLD to a certain extent, which has significant clinical impacts for the detection and treatment of disease progression. However, multi-regional, multi-faceted, multi-center research evidence is required to confirm and consolidate the current conclusions.

## Data Availability

The original contributions presented in the study are included in the article/**Supplementary Material**. Further inquiries can be directed to the corresponding authors.

## Supplementary material

Supplementary Material 1 The published protocol in PROSPERO.

Supplementary Material 2 Databases retrieval strategy.

Supplementary Material 3 The results of GRADE system.

Supplementary Material 4 Forest plot of circulating irisin levels between MAFLD and the healthy control group by different subgroups (Random-Effects Model, SMD). **(A)** Forest plot of circulating irisin levels between MAFLD and the healthy control group by race (Random-Effects Model, SMD). **(B)** Forest plot of circulating irisin levels between MAFLD and the healthy control group by BMI (Random-Effects Model, SMD). **(C)** Forest plot of circulating irisin levels between MAFLD and the healthy control group by age (Random-Effects Model, SMD). **(D)** Forest plot of circulating irisin levels between MAFLD and the healthy control group by HOMA-IR (Random-Effects Model, SMD). **(E)** Forest plot of circulating irisin levels between MAFLD and the healthy control group by NOS score (Random-Effects Model, SMD). **(F)** Forest plot of circulating irisin levels between MAFLD and the healthy control group by severity (Random-Effects Model, SMD). **(G)** Forest plot of circulating irisin levels between mild MAFLD and moderate to severe MAFLD (Random-Effects Model, SMD). **(H)** Forest plot of circulating irisin levels between MAFLD and the healthy control group by whether to merge T2DM or not (Random-Effects Model, SMD). **(I)** Forest plot of circulating irisin levels between MAFLD and the healthy control group by ELISA kits (Random-Effects Model, SMD).

Supplementary Material 5 Figures of meta-regression. **(A)** Result of meta-regression by race. **(B)** Result of meta-regression by BMI. **(C)** Result of meta-regression by Age. **(D)** Result of meta-regression by HOMA-IR. **(E)** Result of meta-regression by NOS score. **(F)** Result of meta-regression by severity. **(G)** Result of meta-regression by FBG. **(H)** Result of meta-regression by ALT. **(I)** Result of meta-regression by AST. **(J)** Result of meta-regression by T2DM.
