# Supplementary figures and images for: Circulating irisin levels in patients with MAFLD: an updated systematic review and meta-analysis
Source: Front Endocrinol (Lausanne). 2024 Dec 17;15:1464951. doi: 10.3389/fendo.2024.1464951 (PMC11686449; doi:10.3389/fendo.2024.1464951)

A

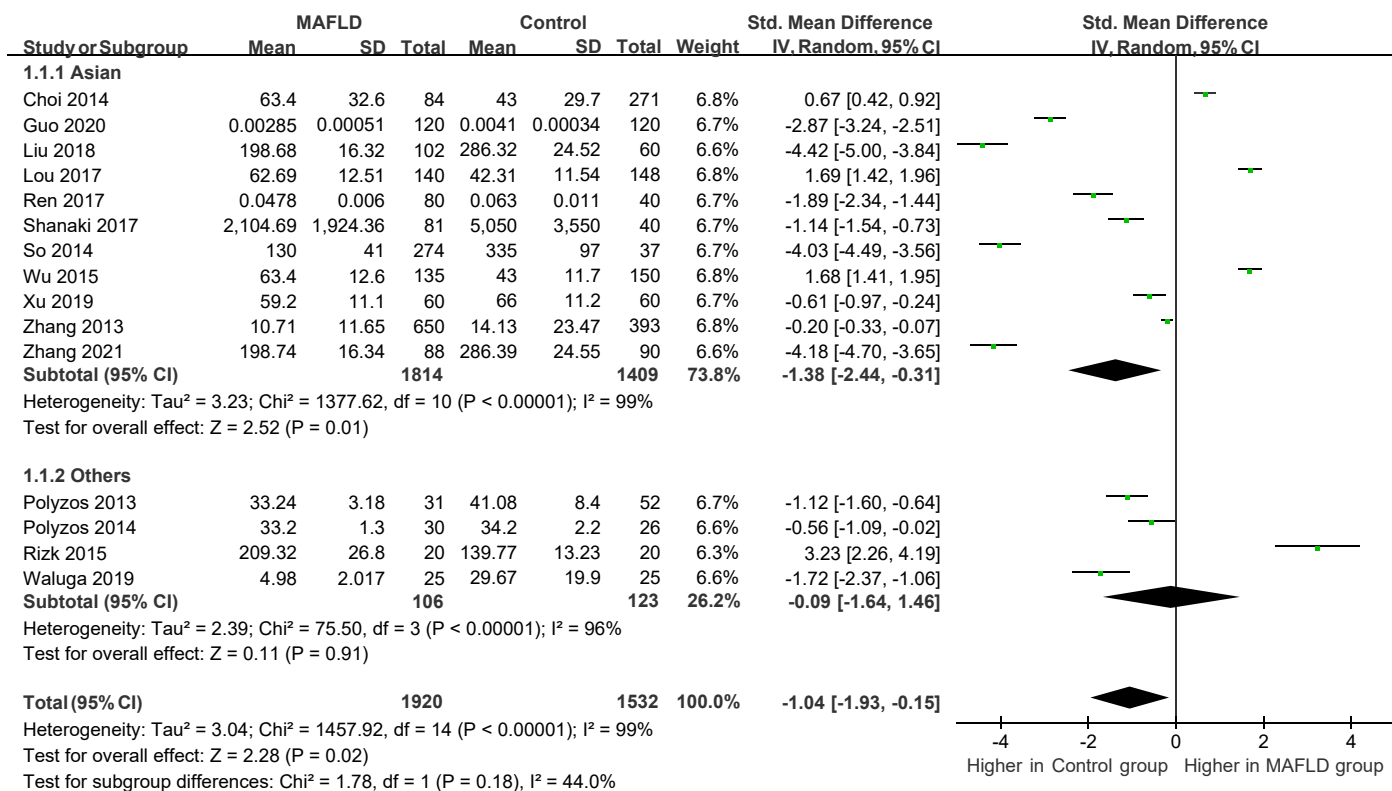

Supplement: Supplementary Material 1 — The published protocol in PROSPERO. [file Image1.pdf]

# B

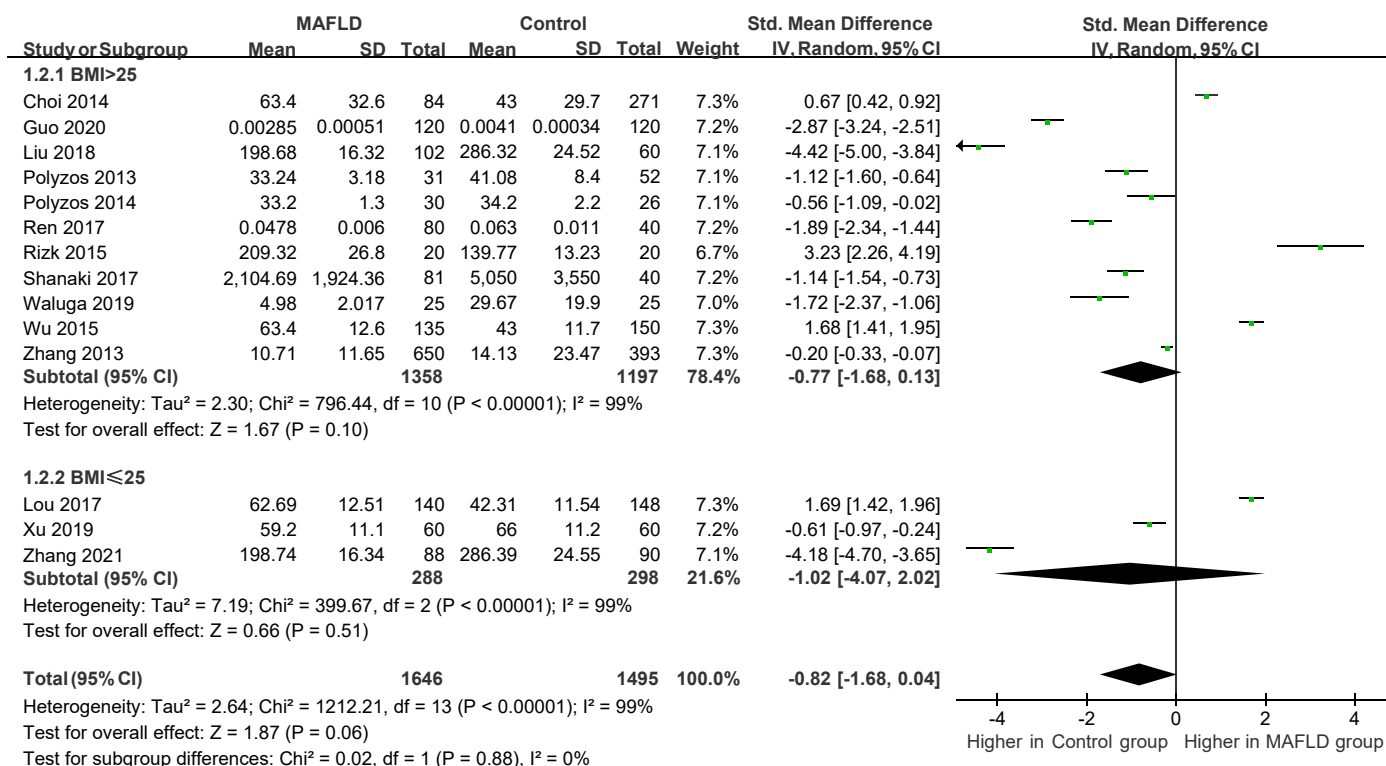

Supplement: Supplementary Material 2 — Databases retrieval strategy. [file Image2.pdf]

C

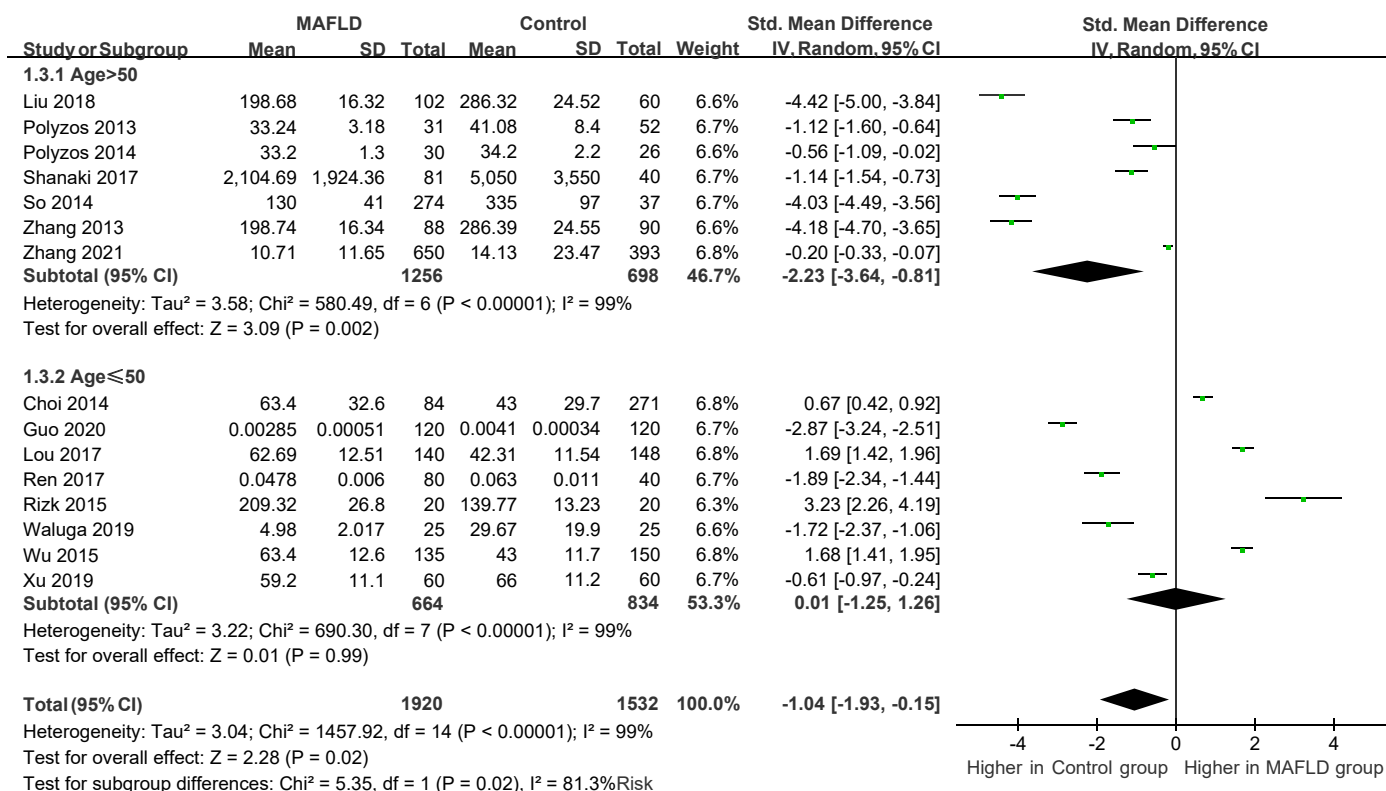

Supplement: Supplementary Material 3 — The results of GRADE system. [file Image3.pdf]

# D

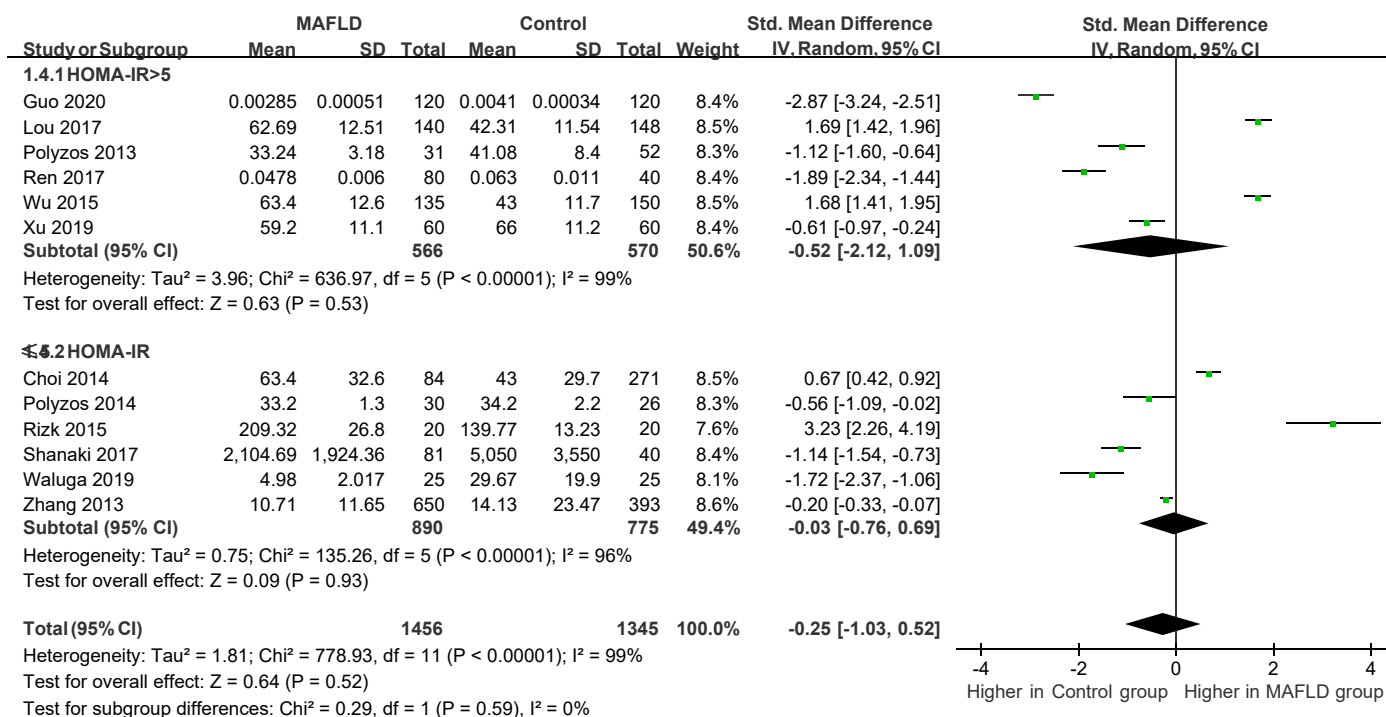

Supplement: Supplementary Material 4 — Forest plot of circulating irisin levels between MAFLD and the healthy control group by different subgroups (Random-Effects Model, SMD). (A) Forest plot of circulating irisin levels between MAFLD and the healthy control group by race (Random-Effects Model, SMD). (B) Forest plot of circulating irisin levels between MAFLD and the healthy control group by BMI (Random-Effects Model, SMD). (C) Forest plot of circulating irisin levels between MAFLD and the healthy control group by age (Random-Effects Model, SMD). (D) Forest plot of circulating irisin levels between MAFLD and the healthy control group by HOMA-IR (Random-Effects Model, SMD). (E) Forest plot of circulating irisin levels between MAFLD and the healthy control group by NOS score (Random-Effects Model, SMD). (F) Forest plot of circulating irisin levels between MAFLD and the healthy control group by severity (Random-Effects Model, SMD). (G) Forest plot of circulating irisin levels between mild MAFLD and moderate to severe MAFLD (Random-Effects Model, SMD). (H) Forest plot of circulating irisin levels between MAFLD and the healthy control group by whether to merge T2DM or not (Random-Effects Model, SMD). (I) Forest plot of circulating irisin levels between MAFLD and the healthy control group by ELISA kits (Random-Effects Model, SMD). [file Image4.pdf]

# E

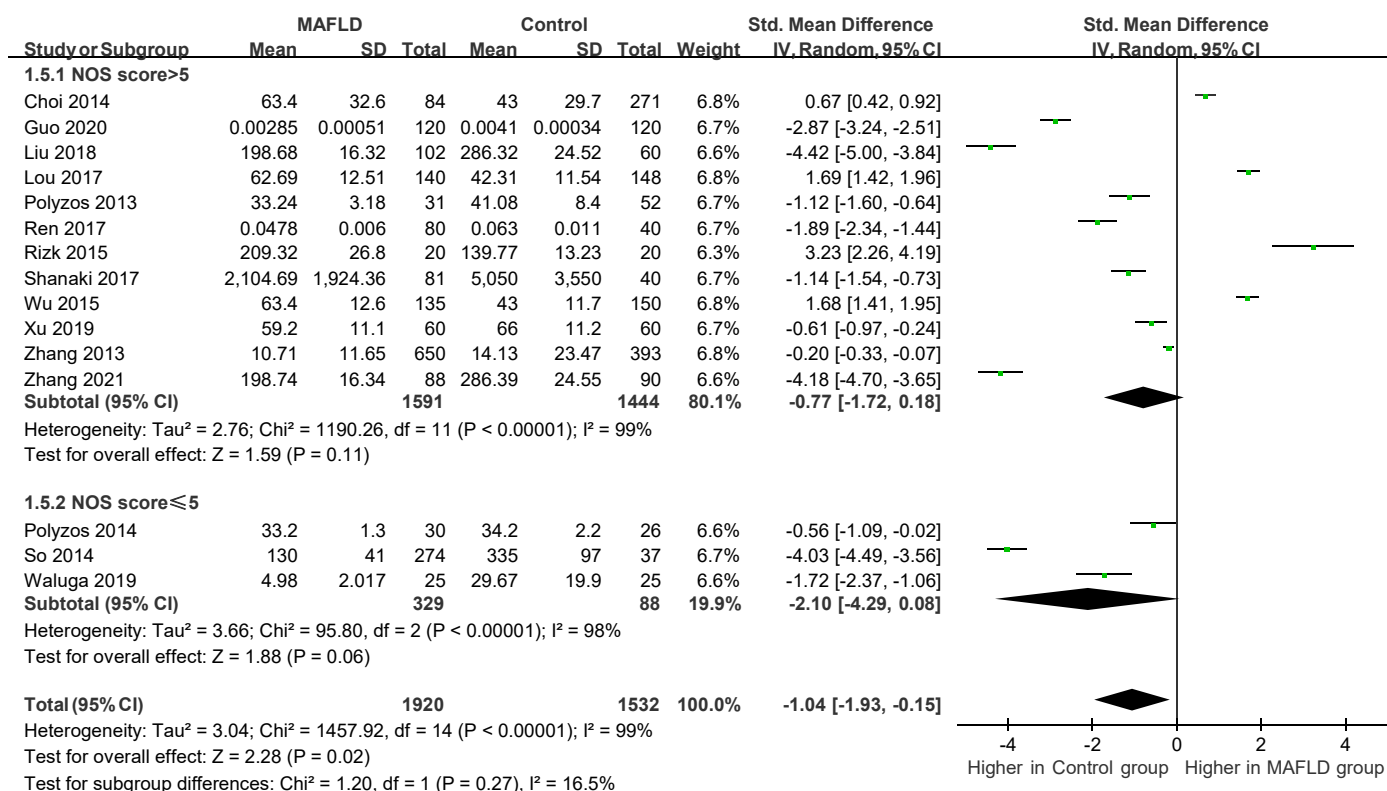

Supplement: Supplementary Material 5 — Figures of meta-regression. (A) Result of meta-regression by race. (B) Result of meta-regression by BMI. (C) Result of meta-regression by Age. (D) Result of meta-regression by HOMA-IR. (E) Result of meta-regression by NOS score. (F) Result of meta-regression by severity. (G) Result of meta-regression by FBG. (H) Result of meta-regression by ALT. (I) Result of meta-regression by AST. (J) Result of meta-regression by T2DM. [file Image5.pdf]

## F

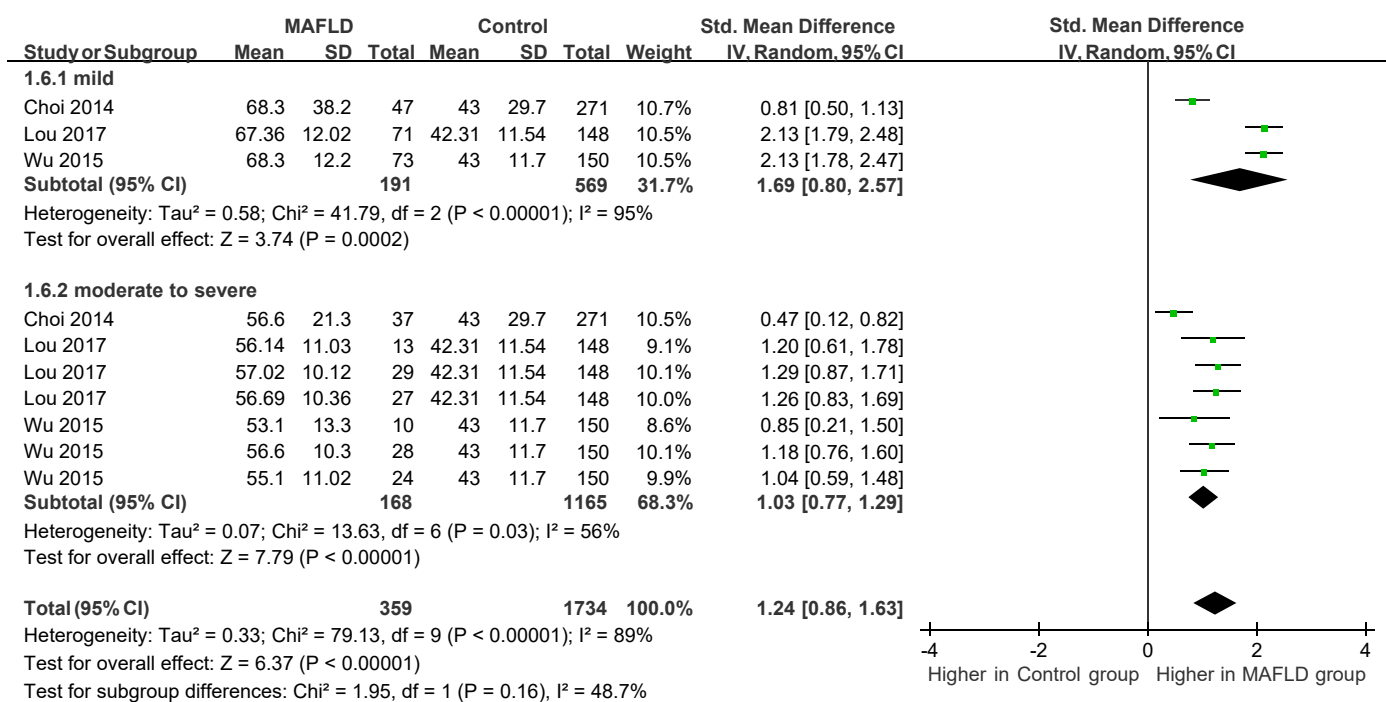

Supplement: Supplementary file 6 [file Image6.pdf]

# G

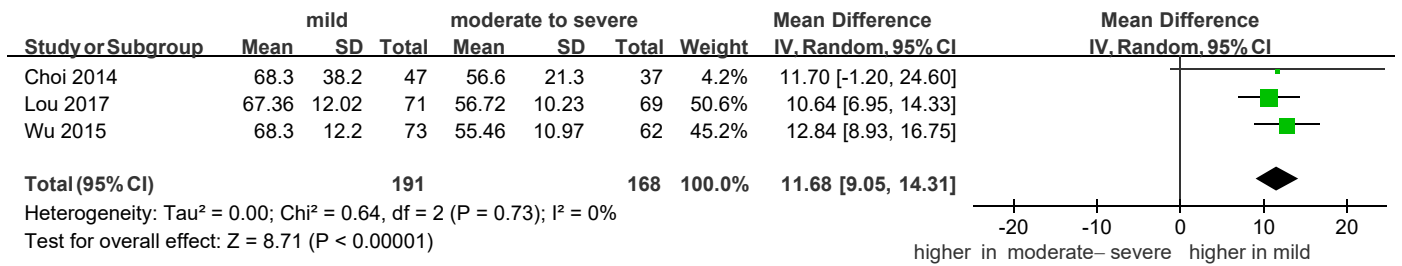

Supplement: Supplementary file 7 [file Image7.pdf]

# H

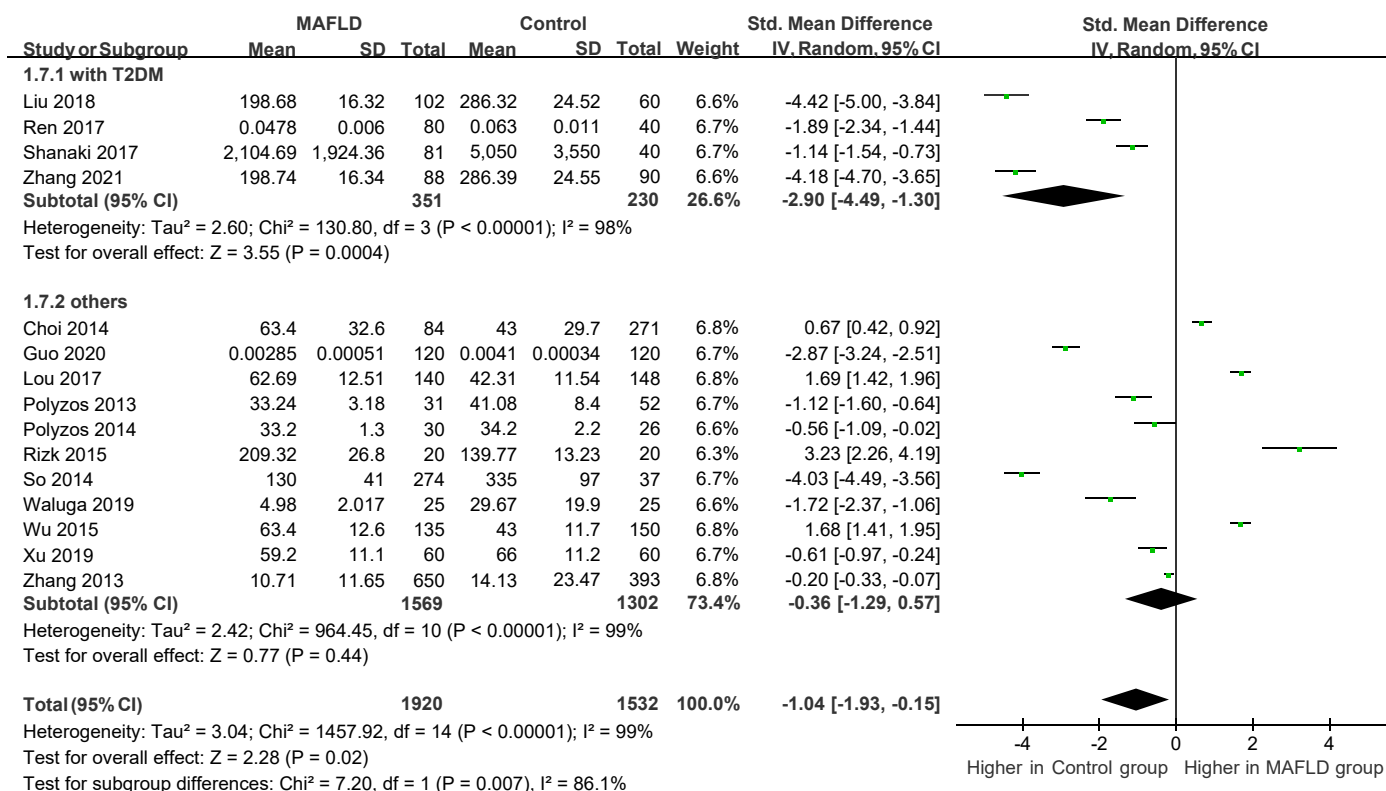

Supplement: Supplementary file 8 [file Image8.pdf]

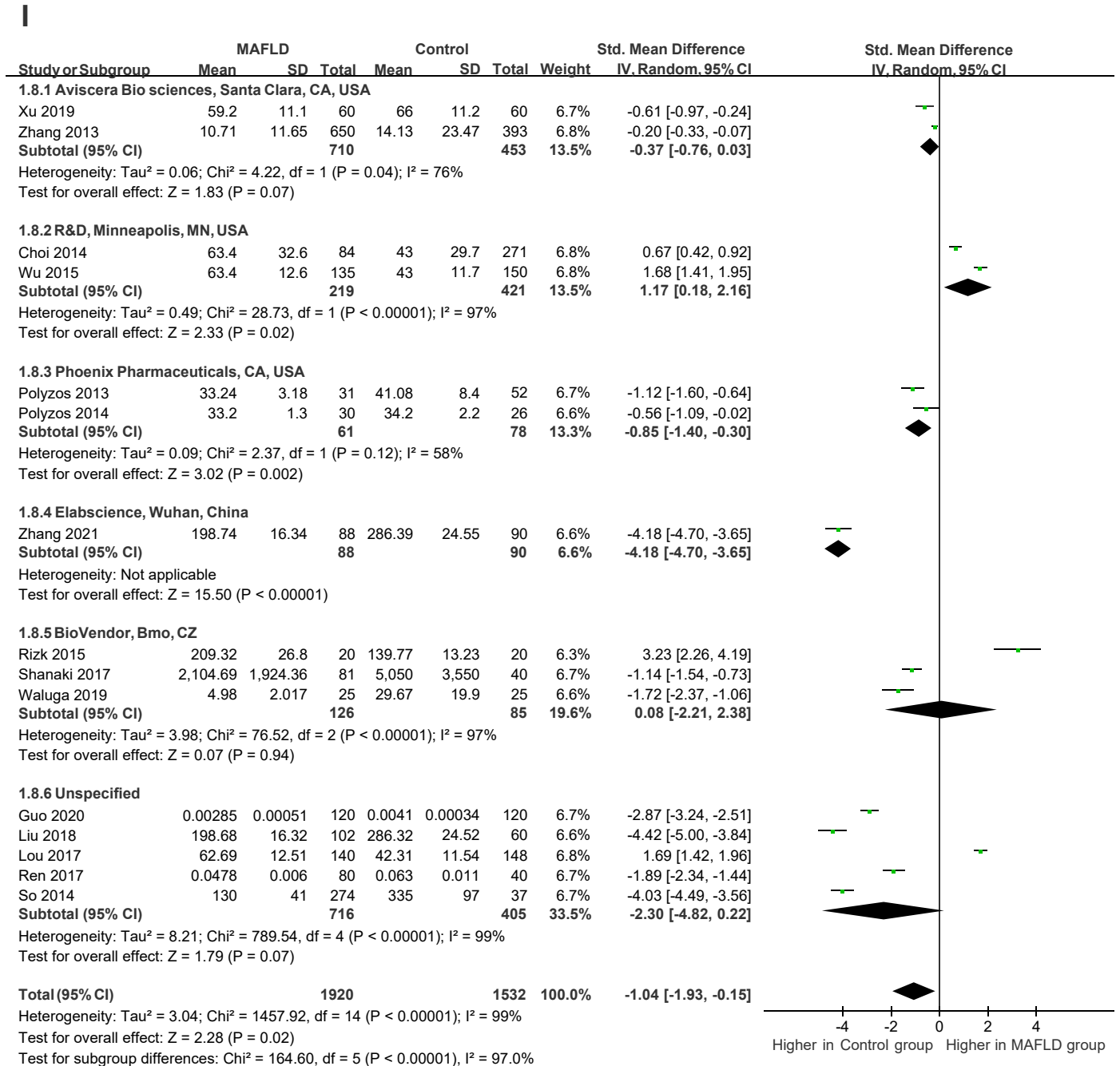

Supplement: Supplementary file 9 [file Image9.pdf]

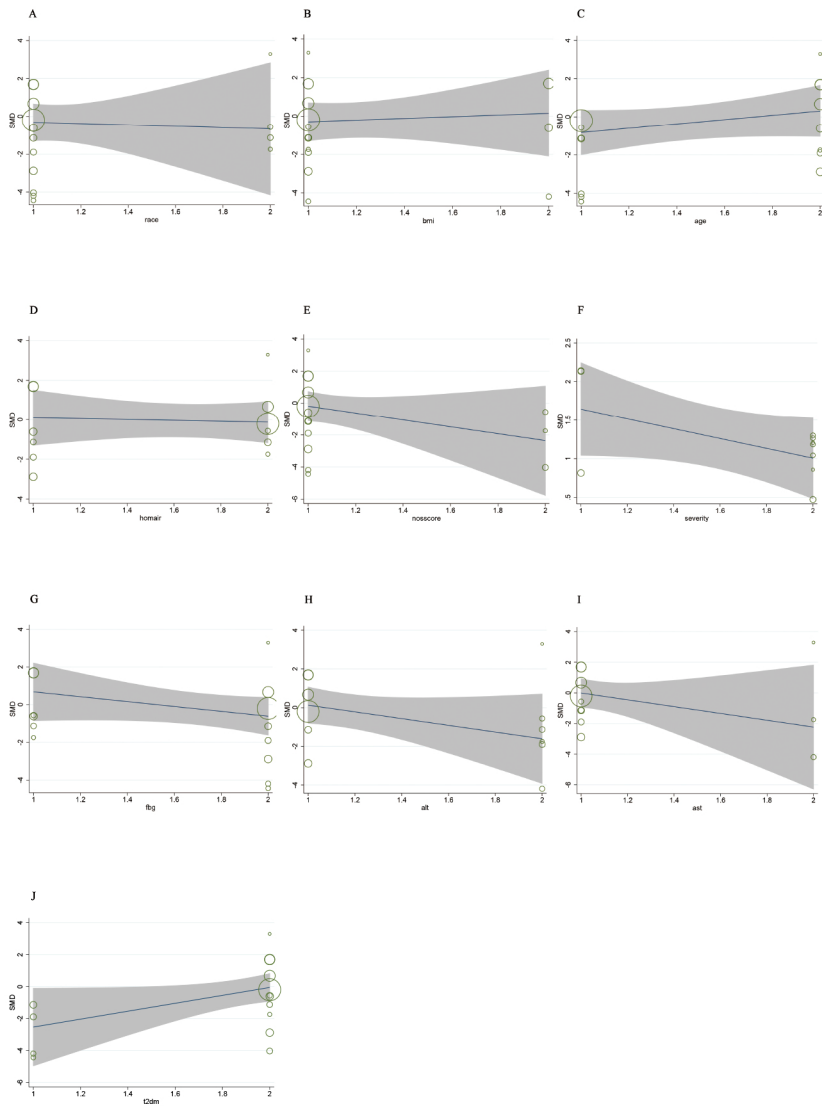

Supplement: Supplementary file 10 [file Image10.pdf]
