# Supplementary material for: Circulating irisin levels in patients with MAFLD: an updated systematic review and meta-analysis
Source: Front Endocrinol (Lausanne). 2024 Dec 17;15:1464951. doi: 10.3389/fendo.2024.1464951 (PMC11686449; doi:10.3389/fendo.2024.1464951)
Supplement: Supplementary file 12 [file Table1.pdf]

Supplementary Material 2

The search is current to 01 August 2024. 1320 Chinese and 228 English were found, for a total of 1548 articles

Databases	Search terms	Number of records
CNKI	FT=('非酒精性脂肪肝'+'非乙醇性脂肪肝'+'非酒精性脂肪性肝病'+'非酒精性脂肪性肝炎'+'非酒精性肝炎'+'脂肪性肝炎'+'非酒精性单纯性脂肪肝'+'代谢相关脂肪性肝病'+'代谢相关性脂肪肝'+'代谢相关性脂肪肝病'+'代谢相关 脂肪性肝炎') and FT=('鸢尾素'+'irisin'+'FNDC5'+'III型纤连蛋白结构域5'+'FRCP2'+'III型纤连蛋白重复包含蛋白 2') 同义词扩展	1174
	(FT= 非酒精性脂肪肝 OR FT= 非乙醇性脂肪肝 OR FT= 非酒精性脂肪性肝病 OR FT= 非酒精性脂肪性肝炎 OR FT= 非酒精性肝炎 OR FT= 脂肪性肝炎 OR FT= 非酒精性单纯性脂肪肝 OR FT= 代谢相关脂肪性肝病OR FT= 代谢相关性脂肪肝 OR FT= 代谢相关性脂肪肝病 OR FT= 代谢 相关脂肪性肝炎) AND (FT= 鸢尾素 OR FT= irisin OR FT= FNDC5 OR FT= III型纤连蛋白结构域5 OR FT= FRCP2 OR FT= III型纤连蛋白重复包含蛋白2) 同义词扩展	1174
Wanfang	全部:(非酒精性脂肪肝 or 非乙醇性脂肪肝 or 非酒精性脂肪性肝病 or 非酒精性脂肪性肝炎 or 非酒精性肝炎 or 脂肪性肝炎 or 非酒精性单纯性脂肪肝 or 代谢相关脂肪性肝病 or 代谢相关性脂肪肝 or 代谢相关性脂 肪肝病 or 代谢相关脂肪性肝炎) and 全部:(鸢尾素 or irisin or FNDC5 orIII型纤连蛋白结构域5 or FRCP2 or III型纤连蛋白重复包含蛋白2) 中英文扩展 主题词扩展	72
VIP	U=(非酒精性脂肪肝 OR 非乙醇性脂肪肝 OR 非酒精性脂肪性肝病 OR 非酒精性脂肪性肝炎 OR 非酒精性肝炎 OR 脂肪性肝炎 OR 非酒精性单纯性脂肪肝 OR 代谢相关脂肪性肝病 OR 代谢相关性脂肪肝 OR 代谢相关性脂肪肝病 OR 代谢相关脂肪性肝炎) AND U=(鸢尾素 OR irisin OR FNDC5 OR III型纤连蛋白结构域5 OR FRCP2 OR III型纤连蛋白重复包含蛋白2)	39
CBM	(非酒精性脂肪肝 or 非乙醇性脂肪肝 or 非酒精性脂肪性肝病 or 非酒精性单纯性脂肪肝 or 非酒精性脂肪性肝炎 or 非酒精性肝炎 or 脂肪性肝炎or 代谢相关脂肪性肝病 or 代谢相关性脂肪肝 or 代谢相关性脂肪肝病 or代谢相关脂肪性肝炎) and (鸢尾素 or irisin or FNDC5 or III型纤连蛋白结构域 5 or FRCP2 or III型纤连蛋白重复包含蛋白 2)	35
Pubmed	Search: (((((((((((((((("Non-alcoholic Fatty Liver Disease"[Mesh Terms])) OR (NASH)) OR (Nonalcoholic fatty liver disease)) OR (Non alcoholic fatty liver disease)) OR (Non alcoholic Fatty Liver Disease)) OR (NAFLD)) OR (Nonalcoholic Fatty Liver Disease)) OR (Nonalcoholic Fatty Liver)) OR (Nonalcoholic Steatohepatitis)) OR (Nonalcoholic Steatohepatitides)) OR (fatty liver OR Liver, Nonalcoholic Fatty)) OR (Steatohepatitides, Nonalcoholic)) OR (Steatohepatitis, Nonalcoholic)) OR (Metabolic Associated Fatty Liver Disease)) OR (MAFLD)) OR (Metabolic dysfunction-associated fatty liver disease)) OR (metabolic associated steatohepatitis)) OR (MASH)) AND ((((((("irisin protein,	73

	zebrafish" [Supplementary Concept]) OR ("FNDC5 protein, human" [Supplementary Concept])) OR (irisin)) OR (FNDC5)) OR (fibronectin type III domain containing protein 5)) OR (Fndc5 protein)) OR (FRCP2 protein))	
Cochrane Library	#1 MeSH descriptor:[Non-alcoholic Fatty Liver Disease] explode all trees<br>#2 fatty liver<br>#3 Liver, Nonalcoholic Fatty<br>#4 Steatohepatitides, Nonalcoholic<br>#5 Steatohepatitis, Nonalcoholic<br>#6 NASH<br>#7 Nonalcoholic fatty liver disease<br>#8 Non alcoholic fatty liver disease<br>#9 Non alcoholic Fatty Liver Disease<br>#10 NAFLD<br>#11 Nonalcoholic Fatty Liver Disease<br>#12 Nonalcoholic Fatty Liver<br>#13 Nonalcoholic Steatohepatitis<br>#14 Nonalcoholic Steatohepatitides<br>#15 Metabolic Associated Fatty Liver Disease<br>#16 MAFLD<br>#17 Metabolic dysfunction-associated fatty liver disease<br>#18 metabolic associated steatohepatitis<br>#19 MASH<br>#20 Metabolically Associated Liver Steatosis<br>#21 #1 OR #2 OR #3 OR #4 OR #5 OR #6 OR #7 OR #8 OR #9 OR #10 OR #11 OR #12 OR #13 OR #14 OR #15 OR #16 OR #17 OR #18 OR #19 OR #20<br>#22 irisin<br>#23 FNDC5<br>#24 fibronectin type III domain containing protein 5<br>#25 Fndc5 protein<br>#26 FRCP2 protein<br>#27 #22 OR #23 OR #24 OR #25 OR #26<br>#28 #21 AND #27	11
EMBASE	#1 'nonalcoholic fatty liver'/exp OR 'nonalcoholic fatty liver'<br>#2 'fatty liver' OR 'liver, nonalcoholic fatty' OR 'steatohepatitides, nonalcoholic' OR 'steatohepatitis, nonalcoholic' OR 'nash' OR 'non alcoholic fatty liver disease' OR 'nafld' OR 'nonalcoholic fatty liver disease' OR 'nonalcoholic fatty liver' OR 'nonalcoholic steatohepatitis' OR 'nonalcoholic steatohepatitides' OR 'Metabolic Associated Fatty Liver Disease' OR 'mafld' OR 'Metabolic dysfunction-associated fatty liver disease' OR 'metabolic associated steatohepatitis' OR 'MASH' OR 'Metabolically Associated Liver Steatosis'<br>#3 'fndc5 protein'/exp OR 'fndc5 protein'<br>#4 'irisin'/exp OR 'irisin' OR 'fibronectin type iii domain containing protein 5'/exp OR 'fibronectin type iii domain containing protein 5'<br>#5 'irisin' OR 'fndc5' OR 'fibronectin type iii domain containing protein 5' OR 'fndc5 protein' OR 'frcp2 protein'<br>#6 #1 OR #2<br>#7 #3 OR #4 OR #5<br>#8 #6 AND #7	144
