# Supplementary material for: Circulating irisin levels in patients with MAFLD: an updated systematic review and meta-analysis
Source: Front Endocrinol (Lausanne). 2024 Dec 17;15:1464951. doi: 10.3389/fendo.2024.1464951 (PMC11686449; doi:10.3389/fendo.2024.1464951)
Supplement: Supplementary file 13 [file Table2.pdf]

GRADE summary of findings table.

**Outcome**	**Participants (studies)**	**Risk of bias**	**Inconsistency**	**Indirectness**	**Imprecision**	**Other considerations**	**Overall certainty of evidence**
Irisin	15 Observational studies	Not serious	Very serious[1]	Not serious	Not serious	Plausible confounding[2]	VERY LOW

[1]Serious inconsistency due to significant heterogeneity(I^2>90%)

[2]All plausible residual confounding would reduce the demonstrate effect. Some of the studies do not match the basic information of two groups, like age, BMI and so on.
